# The Effect of a Single Bout of Resistance Exercise with Blood Flow Restriction on Arterial Stiffness in Older People with Slow Gait Speed: A Pilot Randomized Study

**DOI:** 10.3390/jcdd9030085

**Published:** 2022-03-16

**Authors:** Samuel Amorim, Alexandra Passos Gaspar, Hans Degens, Maysa Seabra Cendoroglo, Fábio Gazelato de Mello Franco, Raphael Mendes Ritti-Dias, Gabriel Grizzo Cucato, Nicholas Rolnick, Luciana Diniz Nagem Janot de Matos

**Affiliations:** 1 Hospital Israelita Albert Einstein, Sao Paulo 05652-900, Brazil; samuel.amorim.s@gmail.com (S.A.); alexandra.gaspar@einstein.br (A.P.G.); fabio.gfranco@einstein.br (F.G.d.M.F.); 2 Research Centre for Musculoskeletal Science & Sports Medicine, Manchester Metropolitan University, Manchester M1 5GD, UK; h.degens@mmu.ac.uk; 3 Division of Geriatrics, Paulista Medical School, The Federal University, Sao Paulo 04020-050, Brazil; maysa.seabra.cendoroglo@gmail.com; 4 Postgraduate Program in Rehabilitation Science, Universidade Nove de Julho, Sao Paulo 01525-000, Brazil; raphaelritti@gmail.com; 5 Faculty of Health and Life sciences, Northumbria University, Newcastle NE1 8ST, UK; gacucato@gmail.com; 6 Department of Health Sciences, Lehman College, City University of New York (CUNY), New York, NY 10468, USA; nick@thebfrpros.com

**Keywords:** blood flow restriction exercise, arterial stiffness, older people

## Abstract

Purpose: Low-intensity resistance exercise with moderate blood-flow restriction (LIRE-BFR) is a new trending form of exercises worldwide. The purpose of this study was to compare the acute effect of a single bout of traditional resistance exercise (TRE) and LIRE-BFR on arterial stiffness in older people with slow gait speeds. Methods: This was a randomized, controlled clinical study. Seventeen older adults (3 men; 14 women; 82 ± 5 years old) completed a session of TRE (*n* = 7) or LIRE-BFR (*n* = 10). At baseline and after 60 min post-exercise, participants were subject to blood pressure measurement, heart rate measurements and a determination of arterial stiffness parameters. Results: There was no significant difference between the TRE and LIRE-BFR group at baseline. Pulse-wave velocity increased in both groups (*p* < 0.05) post-exercise with no between-group differences. Both exercise modalities did not produce any adverse events. The increase in systolic blood pressure, pulse pressure, augmentation pressure and pulse wave velocity (all *p* > 0.05) were similar after both TRE and LIRE-BFR. Conclusion: TRE and LIRE-BFR had similar responses regarding hemodynamic parameters and pulse-wave velocity in older people with slow gait speed. Long-term studies should assess the cardiovascular risk and safety of LIRE-BFR training in this population.

## 1. Introduction

Ageing is associated with functional and structural changes in endothelium, vascular wall and adventitia. One of these changes is arterial stiffening as a consequence of increased collagen deposition, loss/fragmentation of elastin and the formation of advanced glycation-end products [1] and has been recognized as a strong independent predictor of all-cause mortality related to cardiovascular (CV) events [2]. At the same time, the ageing process is associated with a decline in skeletal muscle mass that may at least be partly attributable to vascular dysfunction, particularly in frail older adults [3].

Strength training improves muscular weakness and reduces atrophy to combat frailty and increase quality of life [4]. Yet, older sedentary adults may not tolerate the loads recommended during traditional strength training (>60% 1RM) [5], perpetuating the frailty phenotype. A new form of strength training involving local vascular restriction with low loads (20–50% 1RM) has been shown to improve muscle mass and strength in healthy older people to a similar extent as traditionally recommended high-intensity strength training [6]. Low-intensity blood flow restriction (LIRE-BFR) training has the potential to positively impact load-compromised individuals by allowing them to strength train at lower loads.

In healthy young people, traditional strength training (TRE) at intensities ≥ 60% 1RM induces an acute increase in arterial stiffness that is reflected by an augmentation of the pulse-wave velocity (PWV) [7,8,9,10]. An increase in arterial stiffness is thought to be a result of metaboreflex activation and higher sympathetic activity that causes a rise in blood pressure [11] and an elevated endothelin-1 concentration, leading to vasoconstriction [12]. This is significant, as an increase of just 1.0 m/s in PWV leads to a 12–14% increased risk of cardiovascular events and a 13–15% increase in mortality [13].

There are, however, concerns regarding the safety of LIRE-BFR in older adults as they often suffer from comorbidities whereby LIRE-BFR potentially has a negative impact on vascular structure and function [14]. To overcome this potential negative impact, low loads are often used during LIRE-BFR, that have been shown to elicit similar metabolic stress to that elicited by TRE, and, therefore, is expected to cause a similar, although not more serious, cardiovascular overload [15]. However, the tissue hypoxia in LIRE-BFR likely elicits an increase in the circulating VEGF, stimulating nitric oxide release [16], ultimately inducing vasodilation [17] that may in fact even result in a lower, rather than elevated, rise in peripheral resistance after a LIRE-BFR session [18] compared to TRE strength training. However, no studies have yet been conducted to determine whether an acute session of LIRE-BFR is safe for those who exhibit slow gait speed and the frailty phenotype. This is important as such people are unable to perform TRE and, hence, they may benefit from LIRE-BFR in clinical practice.

While the above observations show the promising acute and longitudinal effects of LIRE-BFR, the clinical applicability and safety of LIRE-BFR for older people with slow gait speed have not yet been established. Therefore, the purpose of this pilot study was to compare the arterial stiffness responses after one exercise session using LIRE-BFR and TRE in older people with a slow gait speed (<0.9 m/s). It was hypothesized that a single bout of TRE could induce an increase in arterial stiffness (as reflected by an increase in PWV) and that LIRE-BFR does not.

## 2. Methods

### 2.1. Study Design

This was a randomized controlled, clinical pilot study to evaluate the acute effects of one session of LIRE-BFR or TRE on arterial stiffness in older people with slow gait speed. Arterial stiffness parameters, blood pressure, and heart rate were measured before and 60 min after the administration of LIRE-BFR or TRE. The study was approved by the hospital ethics and research committee (CAAE: 56798316.4.0000.0071) and registered at www.clinicaltrials.gov (accessed on 10 January 2022) as NCT03272737. All participants provided their informed written consent before enrollment. The study was conducted in accordance with the standards of the Declaration of Helsinki.

### 2.2. Participants

**Figure 1:**
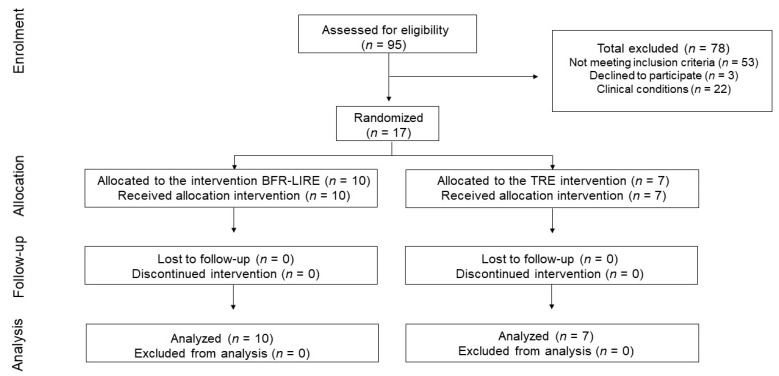
Consort diagram illustrating the number of recruited patients and reasons patients were not included.

Ninety-five older adults (>65 years old) were assessed for study eligibility from a list of patient records from the Albert Einstein Hospital. Inclusion criteria were any adults (both sexes) with gait speed slower than 0.9 m/s over the age of 65 years old. Adults were excluded from the study if their gait speed was greater than 0.9 m/s or any of the following was uncovered in their patient records or clinical examination: uncontrolled diabetes mellitus or peripheral neuropathy, symptomatic peripheral arterial disease, uncontrolled arterial hypertension (BP > 160/100 mmHg), hypercholesterolemia (total cholesterol > 220 mg/dL), infections within the past month, osteoarticular or neurological problems that prevented training, a history of anemia, cerebrovascular disease, or myocardial infarction within the last 6 months, a prior history of a deep-vein thrombosis, current usage of anticoagulants or double antiplatelet agents, a history of smoking within the past 6 months or cognitive impairment (Mini-Mental Status Exam < 24). After screening eligible participants, seventeen older adults were included in the study (Figure 1).

### 2.3. Study Protocol Overview

**Figure 2:**
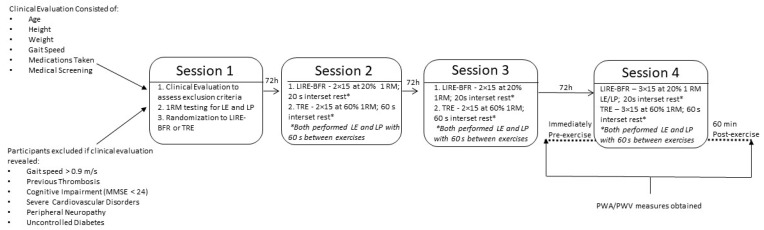
This diagram shows the sequence of events during the pilot study.

Following the initial clinical screening and one-repetition maximum assessment, each participant was randomized into a low intensity blood flow restriction exercise group, performing leg press and leg extensions at 20% 1RM (LIRE-BFR) or traditional strength training (TRE) at 60% 1RM. All participants participated in two familiarization sessions prior to pilot data collection, with each session separated by 72 h. Before and one hour following completion of the selected exercises during the pilot data collection, measures of arterial stiffness were obtained, and the data were used to compare changes between groups. An overview of the study protocol is shown in Figure 2.

#### 2.3.1. Session One—Clinical Evaluation, 1RM Testing and Randomization

##### Clinical Evaluation

Approximately one week before the experimental exercise session, each participant underwent a clinical evaluation conducted by three researchers (SA, AG, LM) to further determine study eligibility. Two researchers (AG, LM) screened participants for previous thrombosis, cognitive impairment via Mini Mental Status Exam (score < 24 indicates impairment), severe cardiovascular disorders, peripheral neuropathy and uncontrolled diabetes mellitus. If participants were found to have any of the conditions, they were subsequently excluded from further study participation. Additionally, body mass (kg), height (m) and a list of current medications were collected during the clinical evaluation. Body mass index (BMI) was calculated for each eligible participant by dividing body mass by height^2^ (m).

Participants that passed the clinical evaluation performed the 4.6 m walk test to assess gait speed [19]. The test was repeated three times with 30 s rest between attempts and the median score was used for analysis. Participants were excluded from further analysis if they exhibited a gait speed of >0.9 m/s [20]. Each participant was advised to continue taking their prescribed medications along with instructions to consume a light meal, drink water, and avoid physical activity and alcohol prior to reporting to the lab for each session. After session one, participants were also told to avoid consuming caffeine, chocolate and tea 24 h prior.

##### One-Repetition (1-RM) Maximum Assessment

Following the clinical evaluation and gait assessment, participants who met the inclusion criteria for the study participated in a 1-RM assessment for the leg press (LP) and leg extension (LE) exercises. The 1-RM assessment was performed for each exercise in accordance with the guidelines of the American Society of Exercise Physiologists for isotonic resistance testing [21]. LP (VR4860, Cybex International Inc., Medway, MA, USA) was performed before LE (VR2, Cybex International Inc., Medway, MA, USA) as this mirrored the experimental design in session 4. Five minutes of rest was allocated after determining the 1-RM in the LP before moving onto the LE.

The testing protocol consisted of a specific warm-up with 50% of the participant’s estimated 1-RM. One minute of rest was given and then each participant performed one set of three repetitions of their estimated 70% 1-RM. After a 3 min rest period, the participant had up to five attempts to achieve their 1-RM. Loads were determined subjectively and a successful repetition was defined as movement of the knee joint from 90° to 0° of flexion in the exercise. If the participant successfully completed the repetition, three minutes of rest were allocated, and a minimal amount of additional weight was added that was judged to be reasonable given the performance of the prior repetition. This process was repeated until a 1-RM was achieved.

##### Randomization

Each included participant was randomized into one of the two groups (LIRE-BFR or TRE) using the website “randomizer.org”(available online: http://www.randomizer.org/ accessed on 4 April 2018). The researchers who performed the experiments before and after the exercise session were blinded to the participant’s group allocation. However, the blood flow restriction specialist who conducted the exercise session was not blinded.

#### 2.3.2. Session Two and Three—Familiarization

Seventy-two hours after inclusion in the study, participants underwent two familiarization sessions (each with 72 h of rest in-between) in accordance with their group allocation. LIRE-BFR performed two sets of 15 repetitions at 20% 1-RM with 20 s of inter-set rest. TRE performed the same sets and repetitions but at 60% 1-RM and 60 s of inter-set rest. Exercise cadence was set at 2 s for the concentric, 2 s for the eccentric and was tracked with a metronome. After performing the LE, participants in both groups were given 60 s of rest before beginning LP [22,23].

##### Determination of Individual Blood Flow Restriction Pressure

Application of LIRE-BFR during the familiarization and pilot data collection was performed with the KAATSU Nano device (KAATSU Global, Los Angles, CA, USA); 5 cm cuff width), an automatic autoregulated BFR device. Applied pressure was determined in accordance with the KAATSU manual guidelines with the participant in sitting and the device on the bilateral proximal thighs. [24] During the LIRE-BFR exercise session, pressures ranged from 150–260 mmHg between the older adults and was applied continuously throughout each set, including the rest periods. Following completion of the exercise, the cuff was deflated to 0 mmHg [25].

#### 2.3.3. Session Four—Pilot Data Collection

##### Exercise Session

After ~72 h following the last familiarization session, participants reported to the lab where baseline measures of arterial stiffness were collected. After one hour, participants exercised in their allocated group with LIRE-BFR performing LP/LE at 3 sets of 15 repetitions with 20 s of inter-set rest with 20% 1RM while TRE performed the same exercise with 60% 1RM and 60 s of inter-set rest [26,27]. Inter-exercise rest was 60 s for each group. LE was performed before LG in both groups and the exact machines and cadences were used as in the familiarization sessions. Participants breathed normally. None of the participants reported pain or discomfort during the exercise in either group.

##### Assessment of Arterial Stiffness

Measures of arterial stiffness were performed before the pilot exercise session and 60 min after its completion. Each participant was positioned in supine in a quiet room with controlled temperature (20–25 °C) for 10 min prior to data collection.

Pulse wave analysis (PWA) and pulse wave velocity (PWV) measurements were performed using applanation tonometry (Sphygmocor; AtCor Medical, Sydney, Australia). Aortic pressure waveforms and blood pressures were derived from the radial artery using a validated transfer function. The aortic pressure waveform was in turn used to calculate augmentation pressure (AP), augmentation index (AIx) and AIx corrected to a heart rate of 75 bpm (AIx75) [28].

The systolic part of the central waveform is defined by two pressure peaks: the first peak (P1) caused by the left ventricular ejection and the second peak (P2) is the reflected wave, where AP is measured as the difference between P2 and P1. The AIx was expressed as the AP, as a percentage of the pulse pressure (PP). The average of three measurements of radial blood pressure of high-quality (operator index > 80%) were used in the analysis [29].

For the PWV analysis, the distance from the carotid artery to the suprasternal notch, and femoral artery to the suprasternal notch were measured using a measuring tape. The PWV was automatically calculated by the Atcor software as the carotid-femoral artery distance divided by the wave travelling time between sites [30]. A simultaneous ECG recording was used to assess heart rate. All post-exercise measurements were done in the LIRE-BFR group when the device was deflated.

##### Statistical Analyses

Continuous variables were expressed as mean ± standard deviation or mean and 95% confidence interval (IC), categorical variables as percentages. The distributions of numerical variables have been evaluated by histograms, QQ plot and Shapiro–Wilk tests. Fisher’s exact tests and Student’s t tests were used to compare groups at baseline. Additionally, model adjustment was verified by an analysis of residuals. To assess exercise-induced changes, generalized linear mixed models were performed on the pre- and post-exercise data in the TRE and LIRE-BFR groups. The sample size and power calculation showed that to detect an 0.5 m/s attenuation in exercise-induced change required 38 people per group at a statistical power of 0.80 and an alpha of 0.05. Even so, such a difference is physiologically not meaningful, particularly when we consider that the difference between groups before exercise was 1 m/s [1].

## 3. Results

Seventeen older adults (14 women; 3 men) above 65 years old completed the entire protocol including the clinical evaluation, group allocation, familiarization sessions and pilot data collection. There was no significant difference between the groups at baseline in any quantitative measures. Participant data collected from the clinical evaluation along with gait speed, 1-RM assessments and reports current medications taken by each participant separated by group allocation are reported in Table 1.

Pilot data suggest similar arterial stiffness responses to LIRE-BFR compared to TRE (Table 2). Some outcome variables did show differences in group (i.e., Aortic DBP, radial DBP and AIx) or time (i.e., Aortic and radial SBP, Aortic and radial pulse pressure, AP, PWV), but the absence of any group x time response indicates this was not influenced by group allocation. Of note, PWV is considered to be the gold standard for arterial stiffness assessment and did not show any between-group differences [31].

## 4. Discussion

This is the first study to determine arterial stiffness responses following acute LIRE-BFR in older people with low gait speed (<0.9 m/s). The main findings of the present study were that an acute bout of TRE or LIRE-BFR induced similar increases in arterial stiffness.

Pulse wave velocity (PWV) is considered the main indicator of arterial stiffness and the most used index because of its high reproducibility and clinical utility. Indirect indicators of arterial stiffness, such as central blood pressure, augmentation pressure (AP), represented by the difference between the second reflected and first peak pressure in the pulse wave [32], and the augmentation index (AIx) are also valuable as an elevated AIx is associated with an increased risk of cardiovascular events [28].

In this study, a slight increase in PWV and augmentation pressure were found after both TRE and LIRE-BFR, but this did not result in any adverse event. Although increases in central arterial stiffness in young and middle-aged men have been observed after traditional resistance exercise [33,34,35], low-intensity TRE has no impact on systemic arterial stiffness (brachial-ankle PWV) in middle-aged, older adults and patients with hypertension [36,37,38]. Additionally, TRE may effectively reduce peripheral and central blood pressures in hypertensive middle-aged and older adults [11].

In terms of central hemodynamic parameters, a previous study in healthy adults demonstrated a reduction in AP and AIx up to 30 min post-LIRE-BFR [39]. The discrepancy between this and our study could be attributable to the high age and frailty of our population. As mentioned in a recent review [40], older people with comorbidities may well have vascular dysfunction and hence this may explain the difference in results between the former and our present study.

There are several mechanisms that contribute to vascular stiffness in old age, including calcium deposition, endothelial dysfunction, and diminished relaxation of vascular smooth muscle cells [41]. Furthermore, oxidative stress impairs endothelial-cell repair and induces vascular smooth muscle cell proliferation, resulting in arterial stiffness [42]. In addition, blood pressure is an important determinant of PWV and vascular stiffness [43] and there is the possibility that different people exhibit different levels of oxidative stress and an exercise-induced increases in blood pressure that result in varying changes in arterial stiffness in response to exercise.

In line with our observation in older adults, Rossow et al. [44] observed an acute post-exercise increase in central hemodynamic parameters (cSBP, cDBP cMAP) in active men and women using a 20% 1RM non-failure leg extension protocol applying both wide (13.5 cm) and narrow (5 cm) cuffs. Interestingly, larger increases in central hemodynamic parameters along with rate pressure product (RPP) were observed in the wide cuff compared to the narrow cuff during exercise, suggesting an increased myocardial demand despite a reduced AIx during and immediately after exercise. In their study, participants using wider cuffs completed fewer repetitions (67 ± 2) compared to those exercising with a narrow cuff (75 ± 0). These results indicate that wide cuffs and repetitions until exhaustion may result in greater increases in central hemodynamic parameters. Thus, the use of narrower cuffs and non-failure exercise may be a good approach in specific populations such as frail older adults and people with vascular disease to attenuate the excessive central hemodynamic responses. Of note, Rossow et al. [44] did not apply personalized pressures to each cuff, potentially augmenting the hemodynamic responses in the wider cuff compared to the narrow cuff condition, requiring further research to make more conclusive statements [45].

In our study, despite an increase in the aortic and radial pulse pressure in both groups, there was no significant increase in AIx after TRE or LIRE-BFR. Some authors reported an increase in AIx [46] after resistance exercise but in young, healthy people with an exercise load of 70% rather than 60% of 1 RM, and a higher training volume. Indeed, recently, it was shown that the increases in blood pressure and AIx are proportional to the exercise intensity and not the duration of exercise [47]. Forde et al. [47] suggested that either central or peripheral vascular changes may alter AIx values in the post-exercise period.

These results suggest that LIRE-BFR might be an alternative method to use and help clinical populations such as frail older adults and patients with cardiovascular diseases, as LIRE-BFR did not further compromise vascular health in these patients who had already vascular impairments [48]. Training studies (e.g., 4+ weeks) may help to elucidate whether LIRE-BFR could improve vascular function and safety to a similar degree as TRE, and if so, whether it provides a training approach for load-compromised, frail older adults to safely strength train. Therefore, long-term training studies should be performed now that feasibility and tolerability to LIRE-BFR has been established as the acute spikes in arterial stiffness measures appear to be similar to that elicited by traditional TRE training. This may provide a novel avenue for clinical application and overcome the lower cardiac rehabilitation efficiency to conventional training in older adults [49].

The current study is not without limitations. Firstly, the sample size in each group (*n* = 10 in LIRE-BFR; *n* = 7 in TRE) is somewhat small, though it was large enough to detect increases in stiffness after a single exercise bout. Another limitation is that there was a large proportion of women in the study, which is attributable to (1) the longer life span of men than women and (2) the fact that women typically walk slower than men at any age, and hence will be over represented when selecting for a low walking speed [50]. Thirdly, arterial stiffness measures were only taken at 60 min post-exercise. Other studies using LIRE-BFR sampled arterial stiffness measures at 0–30 min [39,46]. Future studies should include a larger sample size of participants and more arterial stiffness measures taken at various time intervals post-exercise (i.e., 5, 10, 20 min post-exercise) to better identify the between-group differences and to compare with other studies on LIRE-BFR.

## 5. Conclusions

In conclusion, TRE and LIRE-BFR induced similar increases in acute measures of arterial stiffness, indicating that LIRE-BFR is as safe as TRE for frail older people. Future studies are needed to assess the vascular risk and safety of LIRE-BFR training.

## Figures and Tables

**Table 1:** Characteristics of participants and medications.

	TRE	LIRE-BFR	*p*-Value
Age (years)	82.0 (7.4)	82.8 (5.2)	0.796
Body weight (kg)	67.9 (14.6)	67.3 (12.1)	0.929
Height (m)	1.54 (0.06)	1.55 (0.07)	0.774
Body mass index (kg/m^2^)	28.5 (6.3)	27.8 (3.9)	0.762
Gait speed (m/s)	0.74 (0.11)	0.61 (0.13)	0.052
1RM test—Leg press (lb)	110.0 (62.4)	119.0 (35.1)	0.709
1RM test—Leg extension (lb)	70.0 (25.8)	67.0 (18.9)	0.785
Anti-hypertensives	71.4 (%)	100.0 (%)	0.154
Anti-hypercholesterolemia	42.9 (%)	70.0 (%)	0.350
Anti-depressant	42.9 (%)	60.0 (%)	0.637
Anti-anxiety	0.0 (%)	20.0 (%)	0.485
Anti-psychotic	14.3 (%)	10.0 (%)	0.999
Platelet anti-aggregant	42.9 (%)	30.0 (%)	0.644
Oral Hypoglycemic	57.1 (%)	10.0 (%)	0.101
Dementia	14.3 (%)	10.0 (%)	0.999
Anti-convulsant	14.3 (%)	10.0 (%)	0.999
Anti-parkinson	0.0 (%)	20.0 (%)	0.48

TRE: Traditional resistance exercise (6 women, 1 man); LIRE-BFR (8 women, 2 men): Low intensity resistance exercise with blood flow restriction, results are expressed as mean, standard deviation and (%) percentage of participants; *p* < 0.05.

**Table 2:** Arterial stiffness assessments.

Variables	Pre	Post	Effects—*p*-Value		
			Group × Time	Group	Time
Aortic SBP (mmHg)			0.627	0.255	0.025
TRE	118 (106; 131)	124 (110; 138)			
LIRE-BFR	126 (115; 137)	135 (122; 148)			
Aortic DBP (mmHg)			0.373	0.036	0.945
TRE	63 (57; 68)	61 (56; 67)			
LIRE-BFR	69 (64; 74)	70 (65; 75)			
Aortic pulse pressure (mmHg)			0.790	0.723	0.007
TRE	55 (45; 64)	62 (51; 73)			
LIRE-BFR	56 (48; 65)	65 (55; 75)			
Radial SBP (mmHg)			0.623	0.504	0.017
TRE	129 (117; 142)	135 (121; 150)			
LIRE-BFR	133 (122; 144)	143 (129; 156)			
Radial DBP (mmHg)			0.262	0.024	0.843
TRE	62 (57; 67)	60 (55; 66)			
LIRE-BFR	68 (64; 73)	70 (64; 75)			
Radial MAP (mmHg)			0.437	0.112	0.189
TRE	84 (76; 92)	86 (77; 95)			
LIRE-BFR	91 (84; 98)	96 (87; 104)			
Radial pulse pressure (mmHg)			0.891	0.763	0.009
TRE	66 (56; 76)	75 (63; 86)			
LIRE-BFR	64 (55; 73)	73 (62; 84)			
Augmentation pressure (mmHg)			0.130	0.070	0.005
TRE	19 (13; 26)	22 (16; 29)			
LIRE-BFR	23 (18; 29)	33 (27; 39)			
Pulse wave velocity (m/s)			0.682	0.556	0.031
TRE	11.6 (8.7; 15.5)	12.7 (9.6; 16.8)			
LIRE-BFR	10.6 (8.1; 13.8)	11.2 (8.7; 14.5)			
AIx (%)			0.159	0.037	0.146
TRE	36.3 (27; 45)	36.4 (29; 43)			
LIRE-BFR	41.9 (34; 49)	50.2 (43; 57)			
AIx75 (%)			0.152	0.094	0.209
TRE	31.9 (21; 41)	31.3 (24; 38)			
LIRE-BFR	35.9 (27; 44)	44.3 (37; 51)			

TRE: Traditional resistance exercise (*n* = 7); LIRE-BFR: Low intensity resistance exercise with blood flow restriction (*n* = 10). AIx: augmentation index; AIX75: augmentation index corrected by 75 bpm; DBP: Diastolic blood pressure; SBP: Systolic blood pressure; MAP: Mean arterial pressure. Results are expressed as mean and 95% CI. *p*-value: Interactions between time × group, group and time. For all comparisons *p* < 0.05.

## Data Availability

Available from corresponding author upon reasonable request.

## References

[1] Matthew J., Rossman T.J., Martens C.R., Seals D.R. (2018). Healthy Lifestyle-Based Approaches for Successful Vascular Aging. J. Appl. Physiol..

[2] Vanhees L., Rauch B., Piepoli M., Van Buuren F., Takken T., Bo M. (2012). Importance of characteristics and modalities of physical activity and exercise in the management of cardiovascular health in individuals with cardiovascular disease (Part III). Eur. J. Prev. Cardiol..

[3] Carmel M., McEniery Y., Ian R., Hall A.Q., Ian B., Wilkinson J.R.C. (2005). Normal Vascular Aging: Differential Effects on Wave Reflection and Aortic Pulse Wave Velocity. Aging Vasc. Funct..

[4] Lopez P., Pinto R.S., Radaelli R., Rech A., Grazioli R., Izquierdo M., Cadore E.L. (2018). Benefits of resistance training in physically frail elderly: A systematic review. Aging Clin. Exp. Res..

[5] Nelson M.E., Rejeski W.J., Blair S.N., Duncan P.W., Judge J.O., King A.C., Macera C.A., Castaneda-Sceppa C. (2007). Physical activity and public health in older adults: Recommendation from the American College of Sports Medicine and the American Heart Association. Circulation.

[6] Centner C., Wiegel P., Gollhofer A., König D. (2018). Effects of Blood Flow Restriction Training on Muscular Strength and Hypertrophy in Older Individuals: A Systematic Review and Meta-Analysis. Sports Med..

[7] Grigoriadis G., Rosenberg A.J., Lefferts W.K., Wee S.O., Schroeder E.C., Baynard T. (2020). Similar Effects of Acute Resistance Exercise on Carotid Stiffness in Males and Females. Endoscopy.

[8] Okamoto T., Kobayashi R., Sakamaki-sunaga M. (2017). Effect of Resistance Exercise on Arterial Stiffness during the Follicular and Luteal Phases of the Menstrual Cycle. Int. J. Sports Med..

[9] Thiebaud R.S., Fahs C.A., Rossow L.M., Loenneke J.P., Kim D., Mouser J.G., Beck T.W., Bemben D.A., Larson R.D., Bemben M.G. (2015). Effects of age on arterial stiffness and central blood pressure after an acute bout of resistance exercise. Eur. J. Appl. Physiol..

[10] Yoon E.S., Jung S.J., Cheun S.K. (2010). Effects of Acute Resistance Exercise on Arterial Stiffness in Young Men. Korean Soc. Cardiol..

[11] Figueroa A., Okamoto T., Jaime S.J., Fahs C.A. (2018). Impact of high- and low-intensity resistance training on arterial stiffness and blood pressure in adults across the lifespan: A review. Eur. J. Physiol..

[12] Otsuki T., Maeda S., Iemitsu M., Saito Y., Tanimura Y., Ajisaka R., Miyauchi T. (2007). Vascular endothelium-derived factors and arterial stiffness in strength- and endurance-trained men. Am. J. Physiol. Circ. Physiol..

[13] Vlachopoulos C., Aznaouridis K., Stefanadis C. (2010). Prediction of Cardiovascular Events and All-Cause Mortality With Arterial Stiffness. J. Am. Coll. Cardiol..

[14] Spranger M.D., Krishnan A.C., Levy P.D., O’Leary D.S., Smith S.A. (2015). Blood flow restriction training and the exercise pressor reflex: A call for concern. Am. J. Physiol. Circ. Physiol..

[15] Oliveira M., Meireles K., Marty D., Spranger D.S., O’Leary H.R., Peçanha T. (2020). Clinical safety of blood flow restricted training? A comprehensive review of altered muscle metaboreflex in cardiovascular disease during ischemic exercise. Am. J. Physiol. Circ. Physiol..

[16] Uhlmann S., Friedrichs U., Eichler W., Hoffmann S., Wiedemann P. (2001). Direct Measurement of VEGF-Induced Nitric Oxide Production by Choroidal Endothelial Cells 1. Microvasc. Res..

[17] Diaz M., Parikh V., Ismail S., Maxamed R., Tye E., Austin C., Dew T., Graf B.A., Vanhees L., Degens H. (2019). Nitric Oxide Differential effects of resveratrol on the dilator responses of femoral arteries, ex vivo. Nitric Oxide.

[18] Takano H., Morita T., Iida H., Asada K.-I., Kato M., Uno K., Hirose K., Matsumoto A., Takenaka K., Hirata Y. (2005). Hemodynamic and hormonal responses to a short-term low-intensity resistance exercise with the reduction of muscle blood flow. Eur. J. Appl. Physiol..

[19] Lanziotti S., Neri A.L., Ferrioli E., Lourenço R.A. (2016). Fenótipo de fragilidade: Influência de cada item na determinação da fragilidade em idosos comunitários—Rede Fibra Phenotype of frailty: The influence of each item in determining frailty in community-dwelling elderly—The Fibra Study. Cien. Saud. Colet..

[20] Peel N.M., Kuys S.S., Klein K. (2012). Gait Speed as a Measure in Geriatric Assessment in Clinical Settings: A Systematic Review. J. Gerontol. Ser..

[21] Brown L.E.E.E., Weir J.P. (2001). ASEP Procedures recommendation I: Accurate assessment of muscular strength and power. JEP J. Exerc. Physiol..

[22] Nunes J.P., Grgic J., Cunha P.M., Ribeiro A.S., Schoenfeld B.J., de Salles B.F., Cyrino E.S. (2019). What influence does resistance exercise order have on muscle strength gains and hypertrophy? A systematic review and meta-analysis. Sport Med..

[23] Tomeleri C.M., Nunes J.P., Souza M.F., Gerage A.M., Marcori A., Iarosz K.C., Cardoso-Júnior C.G., Cyrino E.S. (2020). Resistance Exercise Order Does Not Affect the Magnitude and Duration of Postexercise Blood Pressure in Older Women. J. Strength Cond. Res..

[24] KAATSU Global Inc. (2017). Kaatsu Equipment User Manual Including Kaatsu Protocols for Including KAATSU Protocols for.

[25] Weatherholt A.M., VanWye W.R., Lohmann J., Owens J.G. (2019). The Effect of Cuff Width for Determining Limb Occlusion Pressure: A Comparison of Blood Flow Restriction Devices. Int. J. Exerc. Sci..

[26] Shimizu R., Hotta K., Yamamoto S., Matsumoto T., Kamiya K., Kato M., Hamazaki N., Kamekawa D., Akiyama A., Kamada Y. (2016). Low-intensity resistance training with blood flow restriction improves vascular endothelial function and peripheral blood circulation in healthy elderly people. Eur. J. Appl. Physiol..

[27] Libardi C.A., Chacon-Mikahil M.P.T., Cavaglieri C., Tricoli V., Roschel H., Vechin F., Conceição M.S., Ugrinowitsch C. (2015). Effect of concurrent training with blood flow restriction in the elderly. Int. J. Sports Med..

[28] Laurent S., Cockcroft J., Van Bortel L., Boutouyrie P., Giannattasio C., Hayoz D., Pannier B., Vlachopoulos C., Wilkinson I., Struijker-Boudier H. (2006). Expert consensus document on arterial stiffness: Methodological issues and clinical applications. Eur. Heart J..

[29] Siebenhofer A., Kemp C.R.W., Sutton A., Williams B. (1999). The reproducibility of central aortic blood pressure measurements in healthy subjects using applanation tonometry and sphygmocardiography. J. Hum. Hypertens..

[30] Bortel L.M., Van Duprez D., Starmans-kool M.J., Safar M.E., Giannattasio C., Cockcroft J., Kaiser D.R., Thuillez C. (2002). Clinical Apllications of Arterial Stiffness, Task Force III: Recommendations for User Procedures. Am. J. Hypertens..

[31] David M., Malti O., AlGhatrif M., Wright J., Canepa M., Strait J.B. (2014). Pulse Wave Velocity Testing in the Baltimore Longitudinal Study of Aging 2. Central Blood Pressure Measurement Using Pulse Wave Analysis (PWA). J. Vis. Exp..

[32] Wilkinson I.B., Mceniery C.M., John R., Lemogoum D., Leeman M., Degaute J., Van de Borne P., Van Bortel L.M. (2005). Pulse waveform analysis and arterial stiffness: Realism can replace evangelism and scepticism. J. Hypertens..

[33] Miyachi M., Kawano H., Sugawara J., Takahashi K., Hayashi K., Yamazaki K., Tabata I., Tanaka H. (2004). Unfavorable Effects of Resistance Training on Central Arterial Compliance. Circulation.

[34] Kawano H., Tanaka H., Miyachi M., Kawano T. (2006). Resistance training and arterial compliance keeping the benefits while minimizing the stiffening. J. Hypertens..

[35] Okamoto T., Masuhara M., Ikuta K. (2009). Upper but not lower limb resistance training increases arterial stiffness in humans. Eur. J. Appl. Physiol..

[36] Casey D.P., Beck D.T., Braith R.W. (2007). Progressive Resistance Training Without Volume Increases Does Not Alter Arterial Stiffness and Aortic Wave Reflection. Exp. Biol. Med..

[37] Collier S.R., Frechette V., Sandberg K., Schafer P., Ji H., Smulyan H., Fernhall B. (2011). Sex differences in resting hemodynamics and arterial stiffness following 4 weeks of resistance versus aerobic exercise training in individuals with pre-hypertension to stage 1 hypertension. Biol. Sex Differ..

[38] Ho S.S., Radavelli-Bagatini S., Dhaliwal S.S., Hills A.P., Pal S. (2012). Resistance, Aerobic, and Combination Training on Vascular Function in Overweight and Obese Adults. J. Clin. Hypertens..

[39] Figueroa A., Vicil F. (2010). Post-exercise aortic hemodynamic responses to low-intensity resistance exercise with and without vascular occlusion. Scand. J. Med. Sci. Sports.

[40] Nascimento C., Schoenfeld B.J., Prestes J. (2019). Potential Implications of Blood Flow Restriction Exercise on Vascular Health: A Brief Review. Sport Med..

[41] Vatner S.F., Zhang J., Vyzas C., Mishra K., Graham R.M., Vatner D.E. (2021). Vascular Stiffness in Aging and Disease. Front. Physiol..

[42] Tsaia J.P., Hsu B.G. (2021). Arterial stiffness: A brief review. Tzu Chi Med. J..

[43] Meani P., Maloberti A., Sormani P., Colombo G., Giupponi L., Stucchi M., Varrenti M., Vallerio P., Facchetti R., Grassi G. (2018). Determinants of carotid-femoral pulse wave velocity progression in hypertensive patients over a 3.7 years follow-up. Blood Press..

[44] Rossow L.M., Fahs C.A., Sherk V.D., Seo D.-I., Bemben D.A., Bemben M.G. (2011). The effect of acute blood-flow-restricted resistance exercise on postexercise blood pressure. Clin. Physiol. Funct. Imaging.

[45] Patterson S.D., Hughes L., Warmington S., Burr J., Scott B.R., Owens J., Takashi A., Nielsen J.L., Libardi C.A., Laurentino G. (2019). Blood Flow Restriction Exercise: Considerations of Methodology, Application, and Safety. Front. Physiol..

[46] Tai Y.L., Marshall E.M., Glasgow A., Parks J.C., Sensibello L., Kingsley J.D. (2019). Pulse wave reflection responses to bench press with and without practical blood flow restriction. Appl. Physiol. Nutr. Metab..

[47] Forde C., Johnston M., Haberlin C., Breen P., Greenan S., Gissane C., Comyns T., Maher V., Gormley J. (2020). Low Dose Resistance Exercise: A Pilot Study Examining Effects on Blood Pressure and Augmentation Index Between Intensities. High Blood Press. Cardiovasc. Prev..

[48] Kambic T., Novakovi M., Tomažin K., Strojnik V., Borut J. (2019). Blood Flow Restriction Resistance Exercise Improves Muscle Strength and Hemodynamics, but Not Vascular Function in Coronary Artery Disease Patients: A Pilot Randomized Controlled Trial. Front. Physiol..

[49] Bianchi S., Maloberti A., Peretti A., Garatti L., Palazzini M., Occhi L., Bassi I., Sioli S., Biolcati M., Giani V. (2021). Determinants of Functional Improvement After Cardiac Rehabilitation in Acute Coronary Syndrome. High Blood Press. Cardiovasc. Prev..

[50] Guimarães R.M. (2020). Expectativa de vida com e sem multimorbidade entre idosos brasileiros: Pesquisa Nacional de Saúde 2013. Rev. Bras. Estud. Popul..

